# Application value of auxiliary anterolateral hip mini-incision combined with traction-free reduction and internal fixation in complex intertrochanteric fractures in older adult patients

**DOI:** 10.3389/fsurg.2026.1742223

**Published:** 2026-06-11

**Authors:** Yong Gao, Kang Chen, Meihua Xiao, Ming Chen

**Affiliations:** 1 Department of Orthopaedics, The Second People’s Hospital of Pingxiang, Pingxiang, Jiangxi, China; 2 Department of Orthopedics and Traumatology, The First Affiliated Hospital of Nanchang University, Nanchang, Jiangxi, China; 3 Department of Ultrasound, The Second People's Hospital of Pingxiang, Pingxiang, Jiangxi, China

**Keywords:** anterolateral hip incision, complex, internal fixation, proximal femoral nail antirotation, reduction without traction table, therapeutic strategies, trochanteric fractures

## Abstract

**Purpose:**

To evaluate the efficacy of anterolateral hip mini-incision combined with traction-free reduction and internal fixation for older adult with complex intertrochanteric fractures.

**Methods:**

Older adult patients (>60 years) with low-energy osteoporotic intertrochanteric fractures who underwent proximal femoral nail antirotation (PFNA) between 2018 and 2023 were enrolled. A total of 41 patients were randomly assigned to the observation group (*n* = 18, anterolateral mini-incision + traction-free fixation) or control group (*n* = 23, traction table-assisted fixation). Primary outcomes included anesthesia duration and reduction quality (Fogagnolo criteria). Secondary outcomes were operative duration, intraoperative blood loss, fluoroscopic exposures, postoperative Visual Analogue Scale (VAS) scores (3rd/10th days), Harris Hip Score (HHS), incision complications, perioperative mortality, and 6-month adverse events (implant cutout/displacement/breakage).

**Results:**

All patients completed surgery and 6-month follow-up without incision complications or internal fixation failure. The observation group had significantly shorter anesthesia duration (73.17 ± 8.79 vs. 96.22 ± 10.50 min) and less intraoperative blood loss (180 ± 21.2 vs. 210 ± 19.7 mL, both *P* < 0.05). No significant differences were noted in operative duration (62.39 ± 7.52 vs. 58.17 ± 8.68 min), fluoroscopic exposures (15 ± 3.1 vs. 13 ± 2.3), postoperative VAS scores (Day 3: 4.33 ± 0.77 vs. 4.34 ± 0.96; Day 10: 3.89 ± 0.58 vs. 4.17 ± 0.78), reduction excellent-good rate (84% vs. 88%), or HHS excellent-good rate (92% vs. 88%, all *P* > 0.05).

**Conclusion:**

Anterolateral hip mini-incision with traction-free supine reduction and fixation is a reliable technique for older adult intertrochanteric fractures. It obviates traction table dependency, shortens anesthesia duration, reduces anesthetic risks in older adult patients, and achieves satisfactory reduction and hip function recovery without increasing blood loss or fluoroscopic exposure. This approach avoids traction table-related complications and is suitable for institutions lacking traction table facilities.

## Introduction

With the growing aging population, the annual incidence of geriatric fragility intertrochanteric fractures is increasing year by year. Internal fixation using third-generation intramedullary nail techniques, such as PFNA and InterTan, can provide stable fixation for geriatric intertrochanteric fractures (1–3). Reduction with a traction table is simple and widely applied; however, it requires prolonged preoperative preparation, which undoubtedly increases the anesthetic risk for older adult individuals. Moreover, the use of a traction table may lead to complications including perineal hematoma and pain, and adequate traction table equipment is unavailable in some medical institutions.

Traction table-assisted reduction for complex intertrochanteric fractures is a sophisticated procedure that requires repeated adjustment of the traction force and position of the traction frame, placing high demands on the coordination between circulating nurses and radiographers. Studies have reported that 3.6% to 22.9% of intertrochanteric fractures cannot be successfully reduced by conventional closed reduction using a traction table and often require adjunctive incisions to achieve satisfactory reduction (4).

This study presents a technique for reduction and internal fixation of fragility intertrochanteric fractures performed in the supine position without a traction table. With the lower limb able to be positioned freely, combined with a mini anterior-lateral hip incision, this technique allows for accurate manipulation and adjustment of complex deformities in complex intertrochanteric fractures, including rotational malalignment, varus displacement, and inter-fragmentary soft tissue entrapment, yielding satisfactory clinical outcomes. The detailed procedure and results are reported as follows.

## Materials and methods

### Patients data and acquisition

This study was approved by the Ethics Committee of the Second People’s Hospital of Pingxiang, Affiliated to Nanchang Medical College (Approval No. 2021/9). This study enrolled 41 patients with low-energy fragility intertrochanteric fractures at our hospital between January 2018 and December 2023. To eliminate the confounding effects of different anesthetic and internal fixation methods, neuraxial anesthesia was administered to all patients, and proximal femoral nail antirotation (PFNA) was uniformly adopted for internal fixation. The observation group (*n* = 18) underwent PFNA via hip anterolateral mini-incision without traction table assistance; the control group (*n* = 23) received PFNA with traction table aid. Baseline data including gender, age, incidence of deep vein thrombosis (DVT), fracture classification, bone mineral density (BMD) and fracture side were compared between the two groups. All patients were followed up for 6 months postoperatively. The following outcomes were observed: wound healing status, occurrence of wound infection and mortality, as well as complications such as internal fixation protrusion and implant migration or failure. Postoperative indicators including anesthesia duration, operation duration, blood loss, fluoroscopic exposure times and visual analogue scale (VAS) scores were compared between the two groups. The primary outcome measures were anesthesia duration, and reduction quality (Fogagnolo criteria). The secondary outcome measures included incision healing, incision infection rate, perioperative mortality, operative duration, intraoperative blood loss, fluoroscopic exposures, postoperative Visual Analogue Scale (VAS) scores (3rd and 10th days), and hip function (Harris Hip Score [HHS]), Adverse events(Implant cutout, displacement, or breakage)within 6 months postoperatively,were also evaluated.All PFNA internal fixation devices implanted in this study were manufactured and supplied by Shandong Weigao Orthopaedic Device Co., Ltd.

Inclusion criteria: Patients aged >60 years with fragility intertrochanteric fractures treated with PFNA internal fixation under intraspinal anesthesia.Exclusion criteria: Pathological fractures, old fractures, open fractures or periprosthetic fractures; multiple fractures; femoral developmental deformities; and non-intraspinal anesthesia.

### Radiological and clinical evaluation

Intraoperative fracture reduction and internal fixation placement were monitored via real-time C-arm fluoroscopic guidance, with standardized radiological evaluation procedures implemented for the two study groups as detailed below.

Observation Group: The C-arm fluoroscope was positioned adjacent to the operating table on the contralateral side of the operative hip, and its placement was marked on the surgical floor to ensure reproducibility. Metal components of the operating table within the surgical field were removed or retracted to avoid imaging interference. Anteroposterior (AP) radiographs of the ipsilateral hip were acquired by aligning the C-arm with the hip joint; the fluoroscope was then rotated to direct the tube toward the femoral neck at the inguinal region, with no intraoperative rotational impediments confirmed preoperatively. Centered on the femoral neck, near-lateral oblique radiographs were obtained from the greater and lesser trochanteric aspects, respectively, to verify the central positioning of the helical blade within the femoral neck.

**Figure 1:**
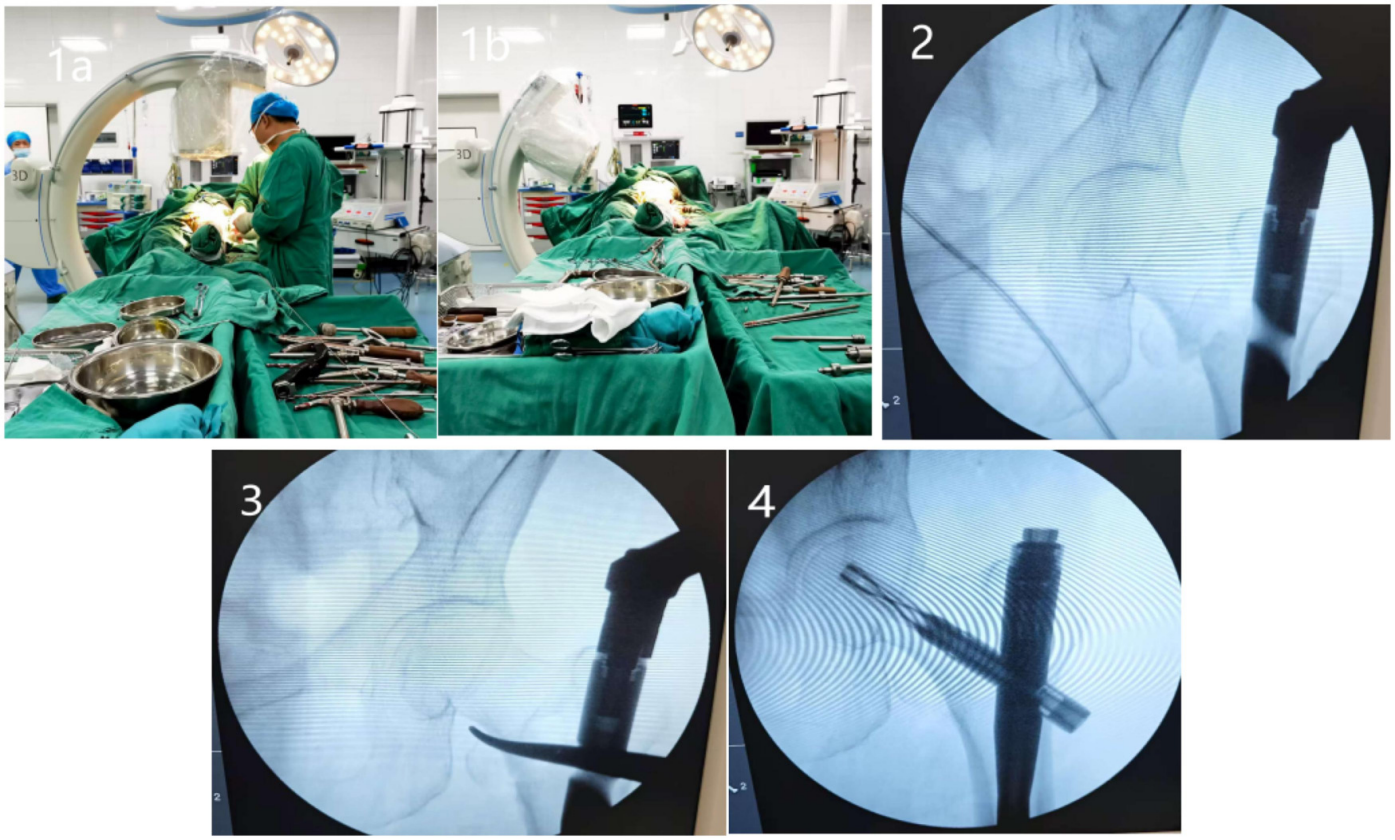
Case 1: **(1a,b)** Intraoperatively, the lateral beam of the C-arm fluoroscope was aligned with the lesser trochanter, and the operating table was tilted to the right. This approach allowed for the acquisition of a near-lateral radiograph. Upon completion of the procedure, a routine frog-leg position x-ray was obtained, which confirmed that the spiral blade was properly seated within the femoral neck. **(2–4)** Following satisfactory manual reduction, mild displacement of the proximal fracture fragment was observed during intramedullary nail insertion. An auxiliary anterior hip incision was then utilized to achieve effective re-reduction, after which internal fixation and locking were performed.

**Figure 2:**
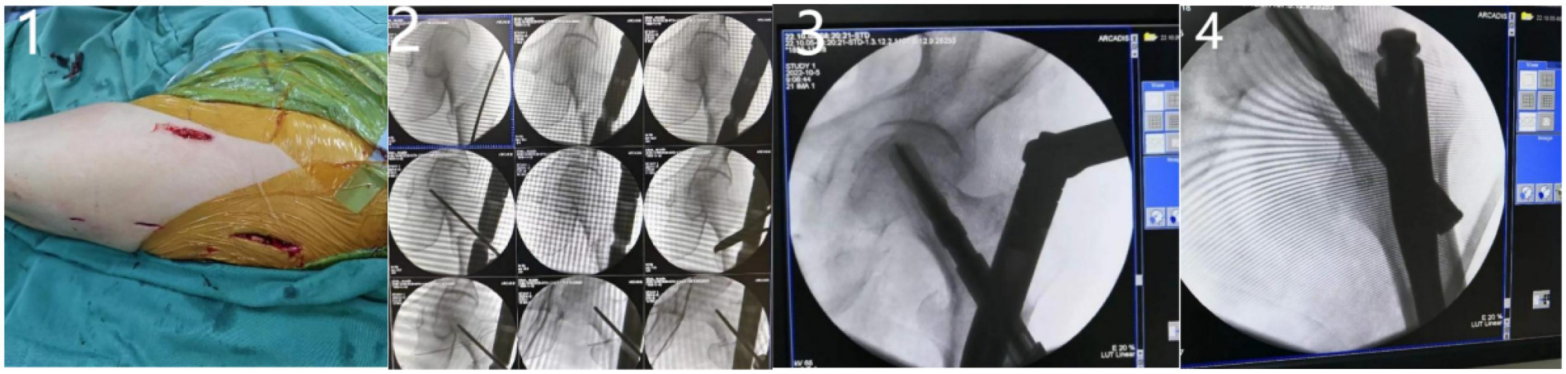
Case 2: **(1)** Intraoperative view showing the auxiliary small incision at the anteroinferior aspect of the hip. **(2–4)** Intraoperative findings of the reduction procedure (performed without a traction table) and corresponding fluoroscopic images. Auxiliary reduction was accomplished via the small anterior hip incision, with the proximal femoral nail antirotation (PFNA) positioned optimally for internal fixation.

Control Group: Patients were placed in a supine position on a surgical traction table, with continuous traction applied to the ipsilateral lower limb for fracture reduction; the contralateral hip was fixed in a flexed, abducted lithotomy position. The C-arm fluoroscope was placed between the bilateral lower extremities, aligned with the ipsilateral hip to obtain AP radiographs, and interleg lateral radiographs (perpendicular to the femoral neck) were acquired subsequently. Fracture reduction was optimized by limited adjustments of the ipsilateral limb position and traction force, based on real-time fluoroscopic findings (Figures 1, 2 of Case 1 and Case 2).

Clinical and Radiological Outcome Assessment Criteria: Intraoperative Satisfactory Reduction Criteria for Intertrochanteric Fractures. Satisfactory intraoperative reduction was defined as: normal neck-shaft angle on AP view with angulation <20° on lateral view; restored anteromedial femoral cortical support (or fracture reduction meeting positive support criteria); and displacement not exceeding the thickness of the medial femoral cortex.

PFNA Internal Fixation Technical Criteria: The head-neck screw was required to be placed in the central or inferior 1/3 region of the femoral neck on both AP and lateral views; the tip-apex distance (TAD), calculated as the sum of AP and lateral TAD values, was required to be ≤25 mm.

### Surgical technique

All patients completed preoperative radiological and laboratory examinations in accordance with the clinical pathway for fragility intertrochanteric fractures. Preoperatively, the patients’ physical conditions were comprehensively evaluated, comorbidities were managed, and obvious surgical contraindications were ruled out. Surgery was performed as early as possible thereafter. The anesthesia modality adopted was intraspinal anesthesia. Both groups received proximal femoral nail antirotation (PFNA) internal fixation for intertrochanteric fracture stabilization.

In observation Group, following disinfection and draping, The patient was positioned supine. Without a traction table, continuous manual traction was applied with the hip flexed to 90°, abduction and external rotation. While maintaining traction, the hip was gradually internally rotated, adducted, and extended. All patients in this group failed to meet the intraoperative imaging reduction criteria as verified by C-arm fluoroscopy; therefore, closed reduction of intertrochanteric fractures was assisted via an anterolateral mini-incision of the hip.

**Figure 3:**
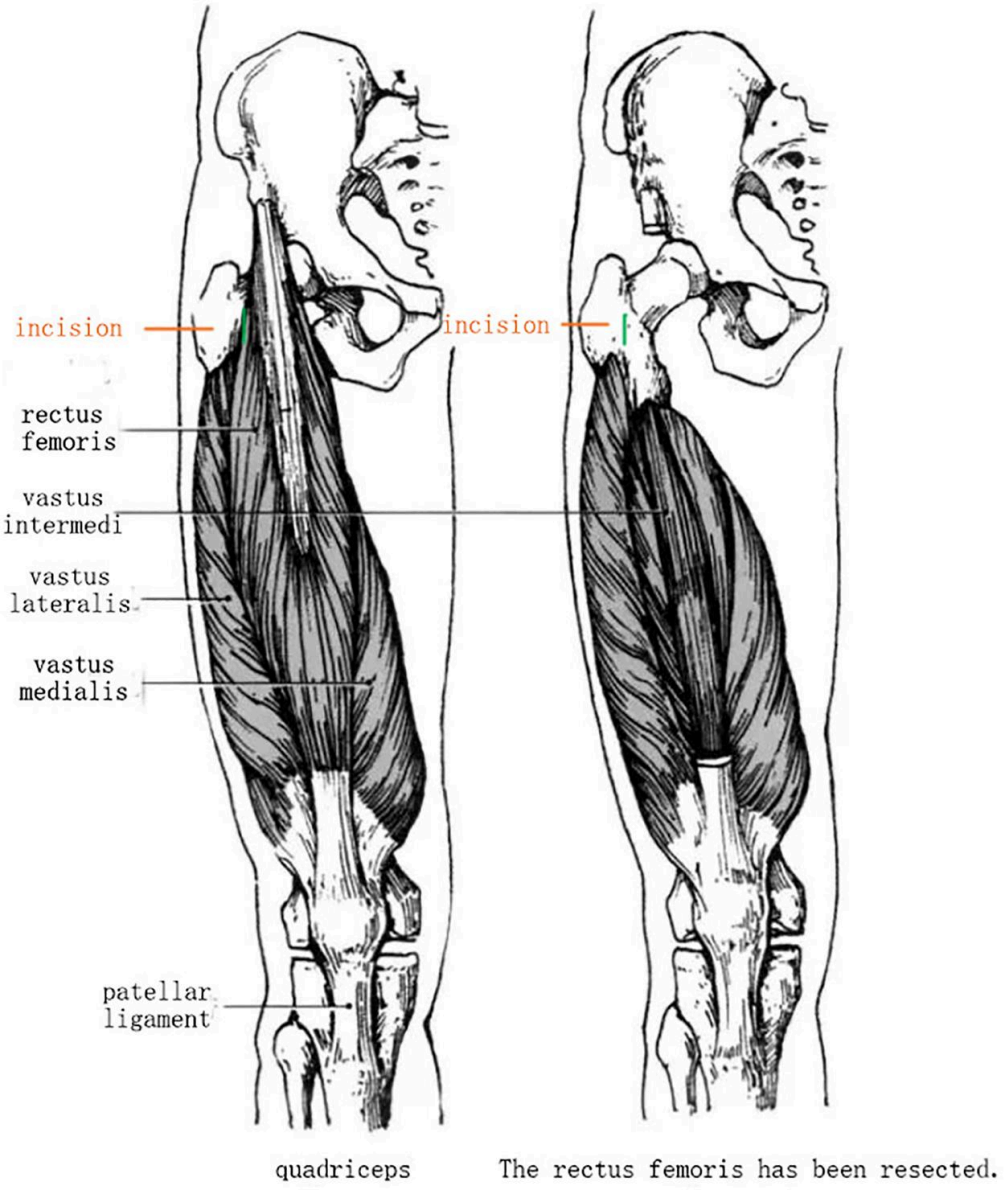
Two anatomical illustrations of the human thigh show the superficial and deep anatomical landmarks corresponding to the auxiliary anterolateral mini-incision of the hip (marked by the green line). The left panel delineates the surgical incision line near the hip, which is located roughly between the greater and lesser trochanters of the femur and superolateral to the lesser trochanter. The right panel presents the regional anatomy of the same thigh after resection of the rectus femoris muscle, demonstrating that this incision provides direct surgical access to the anterior surface of the femoral trochanter.

Compared with the direct anterior approach (DAA) incision, this mini-incision was located more distally and laterally. Under C-arm anteroposterior fluoroscopy, the incision (approximately 1.5–2 cm long) was centered approximately 1.5 cm lateral to the junction of the medial femoral neck and the lesser trochanter (Figure 3). Cut through the skin and subcutaneous tissue,Superficial dissection was performed between the tensor fasciae latae and sartorius, and deep dissection through the rectus femoris down to the femoral surface. Medial palpation along the femoral surface allowed assessment of displacement at the intertrochanteric fracture site.

With double-gloved fingers inserted through the anteromedial hip mini-incision, the displacement of the lesser trochanter, varus angulation, impaction, separation, and bone defect along the medial intertrochanteric border could be palpated. The degree of rotational displacement was roughly estimated by the width of the anterior fracture line. Posteromedial bone loss in intertrochanteric fractures caused anterior angulation, which was also confirmed on preoperative CT reconstruction. For relatively weak strength of hip muscle of older adult patients, longitudinal traction with hip flexion of the lower extremity is sufficient to overcome the shortening displacement of intertrochanteric fractures. Manipulations such as leverage and buttressing via the auxiliary anterolateral mini-incision focus on restoring the integrity of the femoral calcar to ensure normal load transmission, prevent hip varus deformity, and reduce the internal/external rotation discrepancy between the two lower extremities. Once reduction meeting intraoperative criteria was confirmed by C-arm fluoroscopy, the reduced position of the intertrochanteric fracture could be stably maintained, requiring no or only minimal auxiliary traction from an assistant.

Loss of reduction may occur during PFNA implantation, while insertion of the helical blade guide pin or the helical blade into the femoral neck. Intraoperatively, prior to drilling the helical blade guide pin or inserting the helical blade, stabilization of the proximal femoral neck fragment with a finger or a thin periosteal elevator via the anterolateral auxiliary hip incision can effectively prevent separation of the proximal and distal fracture lines, with the proximal femoral neck fragment migrating away from the medial cortex at the lesser trochanter. The entry point should be positioned slightly medial to the apex of the greater trochanter on the anteroposterior fluoroscopic view. On the lateral view, selection of an entry point also affects the anteversion angle of the helical blade in the femoral neck. Distal locking was performed using bicortical fixation. Following completion of PFNA fixation, frog-leg lateral fluoroscopy was obtained to verify the position of the helical blade within the femoral neck. Routine reduction and fixation are not performed for lesser trochanteric avulsion fractures; however, bone grafting (synthetic bone graft or allogeneic bone graft) through the anterolateral mini-hip incision can be performed if there is a significant bone defect at the avulsion site.

In the control group, reduction of intertrochanteric fractures and subsequent PFNA implantation constitute a routine clinical procedure with a traction table. All patients enrolled in this study achieved satisfactory intraoperative reduction of intertrochanteric fractures during traction, reduction, and adjustment on the traction table before disinfection and draping. The C-arm fluoroscope was placed between the two lower extremities, targeting the intertrochanteric fracture site to monitor fracture reduction.

### Statistical analysis

The data obtained in the research were analyzed using the SPSS (Statistical Package for Social Sciences) for Windows 25.0 program. Descriptive statistical methods (number, percentage, mean, and standard deviation) were used while evaluating the data. Power analysis was performed as follows: Type I error was set at *α* = 0.05 and statistical power (1 − *β*) at 0.80. The Bonferroni correction was applied to adjust for multiple comparisons of the three primary outcome measures: anesthesia duration, excellent and good reduction rate according to the Flfafnolo criteria, and excellent and good hip function rate. The adjusted significance level was set at 0.05 ÷ 3 = 0.0167. Effect sizes were reported for all outcome measures. The Kolmogorov-Smirnov test was used to assess the normality of continuous data. For normally distributed data, independent-samples *t*-test was used for between-group comparisons, with Cohen’s d reported as the effect size. For non-normally distributed data, the Mann-Whitney *U*-test was applied, with effect size r reported.Categorical variables were analyzed using the chi-square (*χ*^2^) test or Fisher’s exact test. A *P*-value < 0.05 was considered statistically significant for all comparisons.

## Results

Eliminating the confounding effects of anesthesia and internal fixation modalities on the treatment outcomes of intertrochanteric fractures, The 41 patients were divided into the observation group (*n* = 18) and the control group (*n* = 23). The observation group was treated with PFNA internal fixation for senile fragility intertrochanteric fractures via anterolateral mini-incision of the hip assisted by reduction without a traction table. There were 7 males and 11 females, aged 62–89 years with a mean age of (75.3 ± 6.8) years. The mean dual-energy x-ray absorptiometry (DXA) value was (−3.66 ± 0.85). There were 9 cases on the left side and 9 on the right side. Deep vein thrombosis incidence: 2 cases in 18 cases (11.11%). According to AO classification 2018: 9 cases of type 31-A2.2, 6 cases of type 31-A2.3, and 3 cases of type 31-A3.

The control group was treated with PFNA internal fixation for senile fragility intertrochanteric fractures assisted by a traction table. There were 9 males and 14 females, aged 61–91 years with a mean age of (76.1 ± 7.2) years. Deep vein thrombosis incidence: 4 cases in 23 cases (17.39%). According to AO classification 2018: 11 cases of type 31-A2.2, 8 cases of type 31-A2.3, and 4 cases of type 31-A3. The mean DXA value was (−3.32 ± 1.68). There were 13 cases on the left side and 10 on the right side.

There were no statistically significant differences in general data such as gender, age, Deep vein thrombosis incidence,fracture classification, bone mineral density and fracture side between the two groups (all *P* > 0.05), indicating comparability (Table 1).

**Table 1:** Comparison of parameters between groups.

Parameter		Observation group (*n* = 18) (Mean ± SD)	Control group (*n* = 23) (Mean ± SD)	*p* value
Age		75.33 ± 6.82	75.35 ± 6.83	0.995^a^
Male/Female		7/11	9/14	0.987^c^
Right/Left		9/9	10/13	0.678^c^
DXA value		−3.66 (1.40^e^)	−3.50 (1.20^e^)	0.741^b^
AO/OTA classification	31-A2.2	9	11	1.000^d^
	31-A2.3	6	8	
	31-A3	3	4	
deep vein thrombosis incidence		11.11%	17.39%	0.679^d^

a Independent-samples *t*-test.

b Mann Whitney *U*-test.

c Chi-squared test.

d Fisher’s exact test; SD Standard deviation.

e IR interquartile range.

All patients completed surgery and 6-month follow-up without incision complications or internal fixation failure (implant cut-out, displacement, or breakage). The observation group had significantly shorter anesthesia duration (73.17 ± 8.79 min vs. 96.22 ± 10.50 min) and less intraoperative blood loss (180 ± 21.2 mL vs. 210 ± 19.7 mL) than Group B (both *P* < 0.05). No significant differences were observed in operation duration (62.39 ± 7.52 vs. 58.17 ± 8.68), fluoroscopic exposures (15 ± 3.1 vs. 13 ± 2.3), postoperative VAS scores (Day 3: 4.33 ± 0.77 vs. 4.34 ± 0.96; Day 10: 3.89 ± 0.58 vs. 4.17 ± 0.78) (Table 2).

**Table 2:** Comparison of anesthesia duration, operation duration, blood loss, fluoroscopic exposures, and VAS scores between the two groups.

Group	Anesthesia duration (min)	Operation duration (h)	Intraoperative blood loss (mL)	Number of fluoroscopic exposures	VAS scores	
					Postoperative Day 3 (mean ± SD) (M-IR)	Postoperative Day 10 (mean ± SD) (M-IR)
Observation	73.17 ± 8.79	60.00 (8.25)	180.00 ± 21.21	15.00 ± 3.14	4.33 ± 0.77; (4.00 − 1.00)	3.89 ± 0.58; (3.50 − 1.00)
Control	96.22 ± 10.50	55.00 (15.00)	209.78 ± 19.68	13.22 ± 2.28	4.34 ± 0.96 (5.00 − 1.00)	4.17 ± 0.78 (4.00 − 1.00)
*P* value	0.000^a^	2.354^b^	−4.647^a^	2.026^a^	0.139^b^	0.224^b^
Effect Size	−2.354	0.281	−1.462	0.663	--	0.446

a Independent-samples t-test.

b Mann Whitney *U* test.

SD, standard deviation; M, median; IR, interquartile range.

Fogagnolo Reduction Criteria (first postoperative x-ray review): Excellent: Restoration of normal femoral neck-shaft angle (or mild valgus) on anteroposterior (AP) view; angulation lateral view; fracture reduction alignment > 80%; shortening <5 mm; Good: Meeting any one of the above criteria; Poor: Failing to meet any of the above criteria. According to the Fogagnolo criteria, the excellent and good rates of reduction quality were 88.9% (observation group) and 91.3% (control group) (*P* > 0.05).

Harris Hip Score (last postoperative follow-up visit): Evaluated in four dimensions: pain, function, deformity, and range of motion, with a total score of 100 points. Excellent: 90–100 points; Good: 80–89 points; Fair: 70–79 points; Poor: ≤69 points (5). The excellent and good rates of hip function by the Harris Hip Score were 88.9% (observation group) and 86.96% (control group) (*P* > 0.05) (Table 3).

**Table 3:** Excellent and good rates of reduction quality (Fogagnolo criteria) and hip function (Harris score) between the two groups.

Group	Fogagnolo criteria				Harris hip score				
	Reduction excellent (n)	Reduction good (n)	Reduction poor (n)	Excellent and good rate (%)	Harris excellent (n)	Harris good (n)	Harris fair (n)	Harris poor (n)	Excellent and good rate (%)
Observation group	13	3	2	88.9	11	5	2	0	88.9
Control group	15	6	2	91.3	14	6	2	1	86.96
*P* value	0.886^a^				1.000^a^				

a Fisher’s exact test

## Discussion

Osteoporotic intertrochanteric fracture is a common injury in the elderly population. Intraoperative traction and reduction using a traction table, combined with third-generation intramedullary nails such as the Proximal Femoral Nail Antirotation (PFNA) and InterTan, has become a routine treatment for osteoporotic intertrochanteric fractures in older adult patients. This approach is widely adopted due to its advantages, including lower infection rates, early postoperative ambulation, and less surgical trauma. However, traction tables have several drawbacks, such as prolonged setup time, traction-related complications, and inadequate availability in some medical centers. Moreover, compressive traction on the traction table entails a certain risk for patients with preoperative deep vein thrombosis (DVT) of the lower leg. Accordingly, non-traction-table reduction and fixation techniques have been gradually developed in clinical practice. For example, some authors have reported that intramedullary nailing for femoral intertrochanteric fractures in the lateral decubitus position is a feasible and practical strategy. While the lateral decubitus position allows easier exposure of the nail entry point, skilled surgical techniques are necessary to control rotational deformity and achieve adequate reduction of intertrochanteric fractures (6, 7).

In this study, an anterolateral mini-incision of the hip combined with non-traction-table reduction and internal fixation was applied for the treatment of osteoporotic intertrochanteric fractures in older adult patients. The supine position is more consistent with surgeons’ habitual operating posture.

Eliminating the need for a traction table significantly shortens anesthesia duration. Neuraxial anesthesia exerts less physiological impact on older adult patients and provides more satisfactory lower-extremity muscle relaxation compared with general anesthesia (8), which facilitates manual reduction of femoral intertrochanteric fractures in the supine position. The surgeon can correct the shortening displacement of intertrochanteric fractures by applying traction to the lower extremity with the hip flexed, abducted, and externally rotated. Subsequently, gradual internal rotation, adduction, and extension of the hip allow satisfactory manual reduction of intertrochanteric fractures. Compared with surgery without a traction table, traction table-assisted reduction presents more prominent advantages. Nevertheless, among 1,235 patients with intertrochanteric fractures who underwent traction table-assisted reduction and internal fixation, approximately 15.4% required limited open reduction to achieve satisfactory reduction due to complex intertrochanteric fractures. The main causes of reduction difficulty include complex fracture patterns, predominantly AO classification types A2.1–A2.3 or A3. Studies have shown that comminuted or impacted fractures of the lesser trochanter, lateral wall comminution, loss of medial cortical support, and femoral shaft subsidence all increase reduction difficulty. Reduction difficulty is more likely to occur intraoperatively during supine reduction of intertrochanteric fractures without a traction table. If unaddressed, the advantages of supine reduction and fixation of intertrochanteric fractures without a traction table will be severely diminished. Through repeated surgical practice, we finally adopted mini anterior lateral hip incisions to assist in reduction and fixation of intertrochanteric fractures without a traction table.

The anterior hip approach (direct anterior approach, DAA) is widely used in hip surgery. Previous studies have demonstrated that ultrasound-guided localization of periacetabular neurovascular structures allows safe insertion of Kirschner wires into the femoral head through the anterior incision to assist reduction of complex femoral neck fractures (9). In the present study, the anterolateral hip incision was located slightly more laterally and distally than the standard DAA incision. The anterior hip incision in the DAA approach carries potential risks of femoral nerve palsy (10) and injury to the ascending branch of the lateral femoral circumflex artery. In contrast, the anterolateral hip incision used in this study is positioned more laterally and distally relative to the DAA incision, conferring a lower risk of neurovascular injury. Magnetic resonance imaging (MRI) studies have indicated that the minimally invasive anterior hip (orthopaedic community minimally invasive, OCM) approach utilizes the intermuscular interval between the gluteus medius and tensor fasciae latae, which minimizes muscle damage, improves postoperative function, and offers particular advantages for older adult patients (11). The additional anterolateral incision did not significantly increase blood loss. This may be attributed to the small incision size, which only accommodates one finger; during subsequent reduction, insertion of the finger into the incision exerts a compressive hemostatic effect. Meanwhile, satisfactory reduction facilitates subsequent PFNA implantation for intertrochanteric fractures and shortens anesthesia duration. Compared with the control group, there were no significant increases in operative time, incision pain, incision infection rate, mortality, or deep vein thrombosis (DVT).

For both oblique and reverse oblique intertrochanteric fractures, the core therapeutic objective for osteoporotic intertrochanteric fractures in the elderly is to restore anteromedial cortical support. According to the anteromedial cortical support theory, adequate support can significantly reduce the risks of postoperative neck-shaft angle loss and internal fixation failure (12). This is critical for maintaining postoperative stability (13). In the observation group, direct palpation reduction was performed via the mini-incision, and techniques including leverage were used to accurately correct displacement. This approach is particularly suitable for fractures with anteromedial cortical collapse, effectively restoring the supporting structure, achieving alignment between the femoral head-neck fragment and the distal anteromedial cortex of the femoral shaft, establishing anatomical continuous support, and reducing the risk of hip varus deformity (14). The excellent and good reduction rate according to the Fogagnolo criteria was 84% in the observation group, which was comparable to that in the control group. This indicates that the present technique can achieve reduction quality equivalent to that of traction table-assisted reduction, a key factor for favorable postoperative functional recovery. The auxiliary mini-incision offered an ideal surgical window for restoring the continuity of the medial fracture margin. Direct visualization of the lesser trochanteric fracture through this small incision was neither necessary nor feasible, but the direction of fragment displacement and the malalignment of the medial margin could be clearly identified by digital palpation. In cases with distraction between the proximal and distal fragments, a finger inserted through the incision could easily manipulate the proximal fragment into reduction; subsequent limb adduction allowed impaction of the fragments to achieve temporary stability. For impacted fracture fragments, a long vascular forceps was inserted through the mini-incision under traction, and the impacted proximal fragment could be levered into anatomic reduction with the assistance of limb traction and external rotation. If loss of reduction—most commonly fragment separation—occurred during intramedullary nail insertion, it could be easily corrected using the same technique. During helical blade placement, a finger or vascular forceps was used to buttress the proximal fragment, preventing secondary separation and displacement. Lateral wall disruption is frequently observed in intertrochanteric fractures. Existing literature has documented that low body mass index (BMI) and decreased bone mineral density of the lumbar spine and hip are major risk factors for lateral wall involvement in older adult individuals with intertrochanteric femoral fractures (15).

Poor restoration of the medial column combined with lateral wall disruption impairs the stability of internal fixation. In such cases, a longer and larger-diameter PFNA with a stricter tip-apex distance (TAD) should be used. Some patients may require open plating of the lateral wall (rarely performed) or supplementary anteroposterior screw fixation (16). In this study, the anteromedial mini-incision did not directly reduce lateral wall fractures. However, precise anatomic reduction of the medial column effectively facilitated lateral wall reduction and stability. As supplementary lateral wall fixation significantly prolongs operation time and increases surgical trauma and blood loss, we carefully balanced the additional morbidity against the potential gain in stability. Internal fixation systems with inherent lateral wall reinforcement, such as the novel proximal femoral bionic nail (PFBN), may represent a more favorable choice (17).

Intraoperative manipulation of the C-arm fluoroscope was critical in this study, and obtaining satisfactory lateral images during internal fixation required specific technical skills and coordination. The C-arm fluoroscope allowed clear evaluation of fracture reduction on the anteroposterior view and confirmation that the PFNA main nail was located in the middle-inferior 1/3 of the femoral neck. During insertion of the helical blade guide pin, precise verification that the guide pin was positioned in the center of the femoral neck on the lateral view was essential. The quality of the lateral image depended on the rotation angle of the C-arm; the tube was targeted at the medial femoral neck in the inguinal region to achieve optimal imaging, and the greater trochanteric view was obtained when necessary. In clinical practice, this technique relied on proficient experience in C-arm manipulation. With a certain learning curve and improved surgical teamwork, both the operating time and difficulty of C-arm manipulation were significantly reduced.

The anatomy related to the anterolateral hip incision in this study is uncomplicated, but achieving reduction through this incision requires experience and has a certain learning curve. To minimize potential confounding effects on the outcomes, all procedures in both groups were performed by the same surgical team, with the chief surgeon having more than 15 years of experience in orthopedic surgery. To reduce bias from anesthesia and internal fixation approaches, all patients in both groups received neuraxial anesthesia and were fixed with PFNA, which ensured better comparability.

## Limitations of the study

Baseline data lacked parameters such as comorbidity burden, pre-injury mobility, and body mass index (BMI), which may affect the outcomes. Objective radiographic indicators including tip-apex distance (TAD) and anteversion angle were not measured or compared, although they are critical for evaluating internal fixation stability. Owing to the limited scale of the local hospital, the sample size was relatively small. Large-sample, multicenter, prospective studies are required to further verify the long-term efficacy of this technique.

## Conclusion

Reduction and fixation of femoral intertrochanteric fractures in the supine position without a traction table is a reliable and practical surgical technique. Combined with an anterolateral mini-incision of the hip, it allows satisfactory reduction and fixation of complex femoral intertrochanteric fractures. This technique is not limited by traction table equipment and offers multiple advantages: shorter anesthesia duration, controllable blood loss, no increase in fluoroscopic exposure, operation duration and absence of traction table-related risks and complications. It is particularly suitable for medical institutions without traction tables.

## Data Availability

The raw data supporting the conclusions of this article will be made available by the authors, without undue reservation.
